# Complications of Percutaneous Tracheostomy-Assisting Techniques in Critically Ill Patients: A Systematic Review and Meta-Analysis of Randomized Controlled Trials

**DOI:** 10.3390/jcm14228050

**Published:** 2025-11-13

**Authors:** Olga Grajdieru, Constantin Bodolea, Vlad Moisoiu, Cristina Petrișor, Catalin Constantinescu

**Affiliations:** 1 Department of Anesthesia and Intensive Care, Iuliu Hatieganu University of Medicine and Pharmacy, 400012 Cluj-Napoca, Romania; olgadoinag@yahoo.com (O.G.); constantin.bodolea@umfcluj.ro (C.B.); petrisor.cristina@umfcluj.ro (C.P.); 2 Intensive Care Unit, Emergency Clinical Hospital, 400347 Cluj-Napoca, Romania; 3 Municipal Clinical Hospital, 400139 Cluj-Napoca, Romania; 4 MedFuture Research Center for Advanced Medicine, Iuliu Hatieganu University of Medicine and Pharmacy, 400349 Cluj-Napoca, Romania; vlad.moisoiu@gmail.com

**Keywords:** percutaneous tracheostomy, anatomic landmark-guided, ultrasound-guided, bronchoscopy-guided, intensive care, outcomes, complications, meta-analysis

## Abstract

**Background/Objectives:** Percutaneous dilatational tracheostomy (PDT) is a commonly performed procedure in critically ill patients. Various guidance techniques, including anatomical landmark-guided (ALG), ultrasound-guided (USG) and bronchoscopy-guided (BG), aim to enhance procedural safety and efficacy. This systematic review and meta-analysis aimed to compare the safety and efficacy across ALG, USG, and BG techniques in PDT, focusing on complications and procedure times. **Methods:** A systematic review and meta-analysis of randomized controlled trials (RCTs) was conducted. Studies identified through PubMed, CENTRAL, Scopus, and Web of Science databases up to July 2025 comparing ALG, USG, and BG PDT were included. Primary outcomes were minor and major bleeding, with transient hypoxia, transient hypotension, endotracheal tube cuff puncture, pneumothorax, and procedure time as secondary outcomes. Data were pooled using random-effects models, with risk ratios (RR) and 95% confidence intervals (CI) for complications and mean differences for procedure times. Heterogeneity was assessed using I^2^ statistics, with Bonferroni correction for multiple comparisons. **Results:** This meta-analysis included five RCTs (568 patients) comparing USG vs. ALG, six RCTs (404 patients) comparing USG vs. BG, and five RCTs (448 patients) comparing ALG vs. BG. USG significantly reduced minor bleeding compared to ALG (RR 2.30, 95% CI 1.38–3.84, *p* = 0.001) and BG (RR 0.42, 95% CI 0.20–0.91, *p* = 0.02), and major bleeding compared to ALG (RR 2.62, 95% CI 1.00–6.86, *p* = 0.04). ALG was associated with higher minor bleeding risk than BG (RR 1.81, 95% CI 1.05–3.12, *p* = 0.03). No significant differences were found for transient hypoxia, hypotension, endotracheal tube cuff puncture, or pneumothorax across comparisons, though trends suggested lower hypoxia risk with USG and higher pneumothorax risk with ALG. Procedure times were similar (ALG: 10.4 min, USG: 11.7 min, BG: 12.7 min; *p* = 0.493). Some rare complications, like paratracheal placement and mediastinitis, were too infrequent for analysis. **Conclusions:** USG PDT significantly reduces the risk of minor and major bleeding relative to ALG and minor bleeding compared to BG, without prolonging procedure time. USG and BG show comparable safety for most non-bleeding outcomes. No significant differences in procedure times. Future research should focus on larger RCTs to assess rare complications and explore hybrid USG-BG approaches to optimize PDT safety and efficacy.

## 1. Introduction

Since 1985, when Ciaglia et al. [1] described the percutaneous dilatational tracheostomy (PDT) as a safe and minimally invasive surgical airway procedure that can be performed at the patient’s bedside, it has become a cornerstone in critical care. PDT is increasingly one of the most frequently performed procedures in the intensive care units (ICUs) due to its advantages, including a lower risk of complications compared to surgical tracheostomy, reduced procedural time, and the ability to perform it without transferring the patient to the operating room [2]. The most common indications for PDT are critically ill patients with poor neurological status and those with respiratory failure requiring prolonged mechanical ventilation [3]. Over the years, advancements in techniques have further enhanced the safety and efficacy of PDT. Innovations such as ultrasound-guidance (USG) PDT, bronchoscopy-guided (BG) PDT, and improved dilatational tools have made the procedure more precise and accessible [4,5]. However, despite these advancements, high-quality evidence to determine the superiority of one technique or combination of remains limited, highlighting the need for further research to optimize PDT’s safety and efficacy in clinical practice.

The anatomical landmark-guided (ALG) technique relies on neck landmarks to identify the optimal tracheal insertion site. Nonetheless, this approach has low accuracy in predicting the tracheal puncture site and may result in cranial misplacement [4].

In contrast, the USG PDT employs an ultrasound probe to visualize the trachea and the surrounding neck structures, guiding needle placement. This technique minimizes the risk of injury to pretracheal vascular structures by identifying the correct anatomy and vessel location, and ensuring appropriate tube positioning, avoiding overly high placement [4,6]. USG PDT can be performed using either an out-of-plane or a long-axis in-plane approach. The out-of-plane approach visualizes the distance from the skin to the trachea, tracheal diameter, and neck vasculature, but is limited in assessing tracheal ring spacing. The long-axis in-plane approach provides images of the tracheal rings and cricoid cartilage, facilitating accurate needle placement [7]. Needle visualization depends on the needle type, ultrasound machine performance and operator expertise [8]. Pre-procedural ultrasound examination of the neck has led to changes in the planned puncture site in 15% to 50% of cases [9,10,11].

The BG PDT employs a fiberoptic bronchoscope inserted through the endotracheal tube to visualize the trachea from within. This approach prevents injury to the posterior tracheal wall and assists in accurately identifying the puncture site via transillumination. It also confirms the correct positioning of the needle within the trachea (at the 12 o’clock position) and allows for real-time visualization of the entire procedure, including guidewire insertion, tracheal dilation, cannula placement, and final confirmation of proper cannula positioning [5]. This guidance is especially valuable in patients with difficult airways or distorted anatomy, and may offer advantages in patients with excessive bronchial secretions, and can facilitate post-procedural blood aspiration. Downsides include airway occlusion with auto-PEEP, hypercarbia with a transient increase in intracranial pressure, increased procedure time and costs [12,13]. A study suggested that performing PDT under BG did not reduce the incidence of perioperative complications in critically ill patients, provided the procedure was carried out by experienced physicians and in the absence of anatomical abnormalities [14]. However, this assumption may not always hold, and the term “anatomical abnormalities” encompasses a wide range of conditions. Several studies recommend using bronchoscopy during PDT as a safety precaution [15,16,17]. Two retrospective comparative studies found that the use of bronchoscopy did not result in a significant difference in complication rates between procedures performed with and without bronchoscopic guidance [18,19]. In trauma patients requiring PDT, BG is not routinely required, but it serves as a valuable adjunct in patients with difficult anatomy, obesity or cervical spine fixation [20]. However, a retrospective analysis of 3162 PDT procedures concluded that routine bronchoscopic guidance is unnecessary [21]. It appears that the safety of ALG PDT is comparable to that of BG PDT when performed by experienced practitioners [22], but relying solely on experience should not be the standard for the procedure.

The absence of a standardized protocol for PDT poses a significant challenge, particularly for novice practitioners navigating the complexities of this critical procedure in ICUs. Without clear professional guidelines for training and execution, the choice of technique—ALG, USG, or BG—remains contentious. Each method offers unique advantages, yet their comparative safety and efficacy in critically ill patients are poorly defined, with a wide range of complications.

To address this knowledge gap and determine which guided technique optimizes safety, minimizes complications, and enhances procedural success, we conducted a systematic review and meta-analysis of randomized controlled trials (RCTs). By evaluating the outcomes of these PDT techniques in ICU patients, this study aims to establish an evidence-based standard to guide clinical practice and improve patient outcomes.

## 2. Methods

### 2.1. Study Design

This systematic review and meta-analysis were planned and conducted in accordance with the recommendations of the Cochrane Handbook and the Preferred Reporting Items for Systematic Reviews and Meta-Analyses (PRISMA) 2020 guidelines [23,24,25], which are available in the Supplementary Materials. The study protocol was registered on PROSPERO (CRD420251133660), and we fully adhered to it.

### 2.2. Eligibility Criteria

We included only RCTs in our analysis. The search strategy was constructed based on the PICO (Population, Intervention, Comparator, Outcomes) framework to establish our eligibility criteria [26]. The study population consisted of critically ill patients admitted to the ICUs, requiring PDT, with the intervention being one of the techniques against the other. Our primary outcomes were minor bleeding and major bleeding, whereas transient hypoxia, endotracheal tube cuff puncture, transient hypotension, pneumothorax, tracheal ring fracture, mediastinits and procedure time were our secondary outcomes. Excluded studies included those involving pediatric populations, non-ICU settings, manuscripts reusing patient data from other studies, and studies lacking details about ICU outcomes and complications after PDT.

### 2.3. Information Sources

A systematic literature search was conducted across medical literature databases, including MEDLINE (PubMed), Cochrane Library, Scopus, and Web of Science, covering studies published from 1 January 2000 until 31 July 2025. We also conducted a reference search of the included articles. We acknowledge that Embase was not accessed due to institutional constraints.

### 2.4. Search Strategy

A systematic literature search was undertaken using the databases listed above. The search combined keywords and Medical Subject Headings (MeSH) terms, including:

(“Percutaneous Tracheostomy” [Mesh] OR “Percutaneous Tracheotomy” OR “Percutaneous Dilatational Tracheostomy”) AND (“Intensive Care Units” [Mesh] OR “Critical Care” OR “ICU”) AND

(“Bronchoscopy” [Mesh] OR “Ultrasonography” OR “Ultrasound Guidance” OR “Endoscopic Guidance” OR “Assisting Techniques” OR “Guided”) AND (“Safety” OR “Complications” OR “Mortality” OR “Procedure Duration” OR “Success Rate”)

Hand-searching of references from included studies and relevant reviews was performed until no new studies were identified. Search results were managed using Excel (Microsoft Corporation, Redmond, WA, USA), with duplicate references removed. We focused on randomization, as only RCTs were selected.

### 2.5. Screening, Selection and Data Extraction

Following the systematic literature search, articles were imported into an Excel Spreadsheet File (Office 365, Microsoft, Redmond, WA, USA), where duplicates were excluded. The literature search was undertaken by two independent reviewers (O.G. and C.C.) who screened the abstracts, followed by the full texts. In the event of disagreements, consensus was reached through discussion with a third reviewer (C.B). Cohen’s kappa coefficient was computed at both selection levels to assess inter-reviewer agreement [27]. Data extracted from each study included: the first author’s name and publication year, study design, geographic location, total number of participants overall and in each group (ALG, USG, and BG), baseline demographics such as sex and age, procedure duration, and the number of patients experiencing the following complications: minor bleeding, major bleeding, transient hypoxia, transient hypotension, endotracheal tube cuff puncture, pneumothorax, tracheal ring fracture, paratracheal placement, mediastinitis, subcutaneous emphysema, and procedure failure.

Definitions of outcomes of interest:

(1) Minor complications:
  (a) Minor bleeding: bleeding controlled with digital compression, without hemodynamic instability and without the need for surgical revision or transfusion.
  (b) Transient hypoxia: oxygen desaturation during the procedure, defined as SpO_2_ < 90% but ≥85%.
  (c) Transient hypotension: a decrease in blood pressure requiring fluid resuscitation with <1000 mL, without initiation or escalation of inotropic support.
  (d) Barotrauma: occurrence of subcutaneous emphysema.
  (e) Tracheal ring rupture: disruption of a tracheal cartilage ring at any stage of the procedure, recorded only in cases performed under endoscopic guidance.
  (f) Technical complications without clinical repercussions: isolated events such as endotracheal tube cuff puncture, difficulty in cannula insertion, or inability to complete the procedure, provided these events do not result in desaturation or airway loss.
(2) Major complications:
  (a) Major bleeding: bleeding leading to hemodynamic instability and/or requiring surgical revision and/or blood transfusion.
  (b) False passage: creation of a tract resulting in tracheal injury, mediastinal emphysema, or oxygen desaturation (SpO_2_ < 85%).
  (c) Barotrauma: occurrence of pneumothorax or mediastinal emphysema.
  (d) Technical complications with clinical repercussions: events such as endotracheal tube cuff puncture, difficulty in cannula insertion, or inability to complete the procedure, when associated with adverse outcomes, including desaturation, airway loss, or severe complications necessitating a change in management strategy.

### 2.6. Risk of Bias Assessment and Quality of Evidence

To assess the methodological quality of each trial, two independent authors (O.G. and C.C.) evaluated the risk of bias, with a third author (C.P.) resolving disagreements, using the Cochrane Risk of Bias 2 (RoB 2) tool recommended by the Cochrane Handbook [28]. The sources of bias evaluated included the randomization process, deviations from intended interventions, missing outcome data, measurement of the outcome, and selection of the reported result. The Robvis (Risk-Of-Bias Visualization version 2.0) tool was used to create risk-of-bias plots [29]. The quality of evidence was assessed using the GRADE (Grading of Recommendations, Assessment, Development, and Evaluation) [30] approach, considering risk of bias, inconsistency, indirectness, imprecision and publication bias.

### 2.7. Statistical Synthesis

The minimum number of RCTs needed to perform the meta-analysis was three. The meta-analysis between ALG and USG PDT included five RCTs, the analysis comparing USG and BG PDT included six RCTs, and the analysis between ALG and BG PDT included five RCTs. Statistical analyses were performed using statistical software R Studio (version 2025.05.1+513) with meta package and Stata software (version 19.5) to conduct meta-analyses with forest plots and meta-analyses of proportions. Risk ratios (RR) with 95% confidence intervals (CI) were used to measure effect size for binary outcomes. To calculate study-specific and pooled RR, the total number of patients in the intervention and comparator groups was extracted separately from the studies. We reported the results as the risk of an event of interest in the intervention group versus the risk of an event of interest in the comparator group. For continuous outcomes (procedure duration), we extracted the mean and standard deviation (SD) from each study. The study by Gobatto et al. [31] reported the procedure time using median and interquartile range (IQR), so we estimated the mean and SD to facilitate inclusion in the meta-analysis. The mean was approximated as the average of the first quartile (Q1), median, and third quartile (Q3), using the formula: Mean ≈ (Q1 + Median + Q3)/3. The SD was estimated using the formula: SD ≈ (Q3 − Q1)/1.35, as described by Wan et al. [32]. This method assumes approximately symmetric data and is widely used for meta-analyses when only median and IQR are reported. All calculations were performed to ensure compatibility with meta-analytic models requiring mean and SD.

To account for potential heterogeneity across studies, we used the DerSimonian-Laird random-effects model to pool effect sizes [33]. Heterogeneity was assessed using Higgins and Thompson's I*^2^* statistics. Substantial heterogeneity was assumed if the I^2^ value was greater than 50% [34].

All meta-analyses used the Mantel-Haenszel method for the common effect model and the inverse variance method for the random effects model, with RR as the summary measure [35]. Results were considered statistically significant if the 95% CI did not include the null value. We summarized the findings for the meta-analysis in forest plots. In forest plots, for a cell count of zero, the RR of each study with 95% CI was calculated by adding 0.5 as a continuity correction, applied solely for forest plot calculations.

Pooled proportions of complications (minor bleeding, major bleeding, transient hypotension, transient hypoxia, and endotracheal cuff puncture) for the three tracheostomy guidance techniques were estimated using R with the meta, readxl, and ggplot2 packages. Combined rates of complications were calculated using the metaprop function. We initially used generalized linear mixed model (GLMM) to adjust for between-study differences, switching to a simpler approach (inverse variance) if needed. We chose a model that accounts for study variations to estimate the rates and their 95% CI. Results were converted back to percentages for better interpretability.

Pairwise proportion tests were conducted for each complication using Fisher’s exact test for low event counts (<10), and chi-squared tests for higher event counts. *p*-values were adjusted for multiple comparisons (three per complication) using the Bonferroni method to control the family-wise error rate. To compare pooled mean procedure times across the three PDT techniques, pairwise z-tests and a Wald-type test were conducted using R. Pairwise comparisons assessed differences in pooled means, with standard errors derived from 95% CI. *p*-values were adjusted using the Bonferroni method to account for multiple comparisons (three pairs). A Wald-type test evaluated overall differences across all techniques using a chi-squared statistic based on the variance-covariance matrix of the pooled estimates. Statistical significance was assessed at α = 0.05.

Publication bias in small studies was evaluated through visual inspection of funnel plots supported by Egger’s and Begg’s tests and by calculating *p*-values using the Harbord test for RR [36]. Potential publication bias was considered present if the *p*-value was less than 0.10, acknowledging that these tests may lack sufficient power to differentiate between random variation and actual asymmetry.

### 2.8. Ethical Approval

For this systematic review and meta-analysis, no ethical approval was required, as all the articles were already published in peer-reviewed journals. The data used in this study is available in the full-text articles included in this systematic review and meta-analysis. We did not include new patients in our study design, conduct, or interpretation.

## 3. Results

### 3.1. Search and Selection

**Figure 1:**
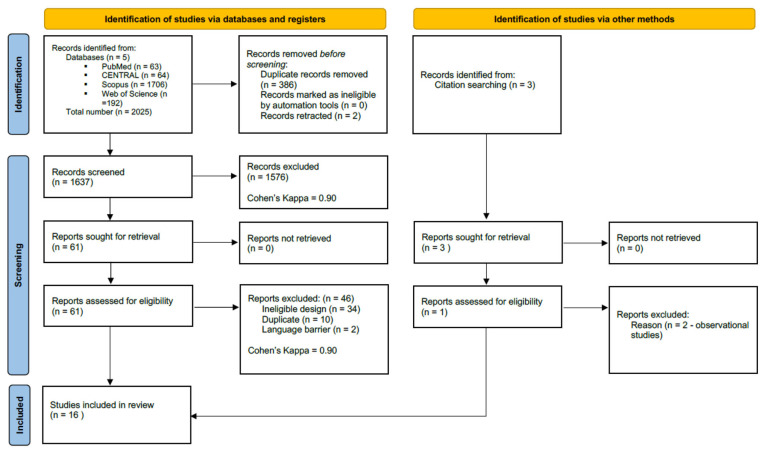
PRISMA flowchart.

In total, 2025 records were identified across the four databases, 63 via PubMed, 64 in CENTRAL, 1706 in Scopus, and 192 from Web of Science. After duplicates removal, 1637 records remained for title and abstract screening. A total of 61 studies were assessed for full-text eligibility, of which 46 were excluded (we searched only for RCTs). In addition, we identified 3 records through citation chasing, but only one study was retrieved and deemed eligible for data extraction. More details on our search and selection process are provided in the PRISMA flowchart (Figure 1).

### 3.2. Basic Characteristics of Studies Included

This meta-analysis synthesized data from three sets of studies, all RCTs, comparing complications of PDT using USG versus ALG, USG versus BG, and ALG versus BG techniques. The first set, comparing USG and ALG, included five studies [7,37,38,39,40] with 568 patients (291 ALG, 277 USG). The second set, comparing USG and BG PDT, included six studies [9,31,41,42,43,44] with 404 patients (203 USG, 201 BG). The last set, comparing ALG and BG PDT, included five studies [22,45,46,47,48] comprising 448 patients (225 USG, 223 BG). More information about the studies is found in Table 1.

### 3.3. Statistical Results

(a) Anatomical landmark vs. Ultrasound-Guided PDT

**Figure 2:**
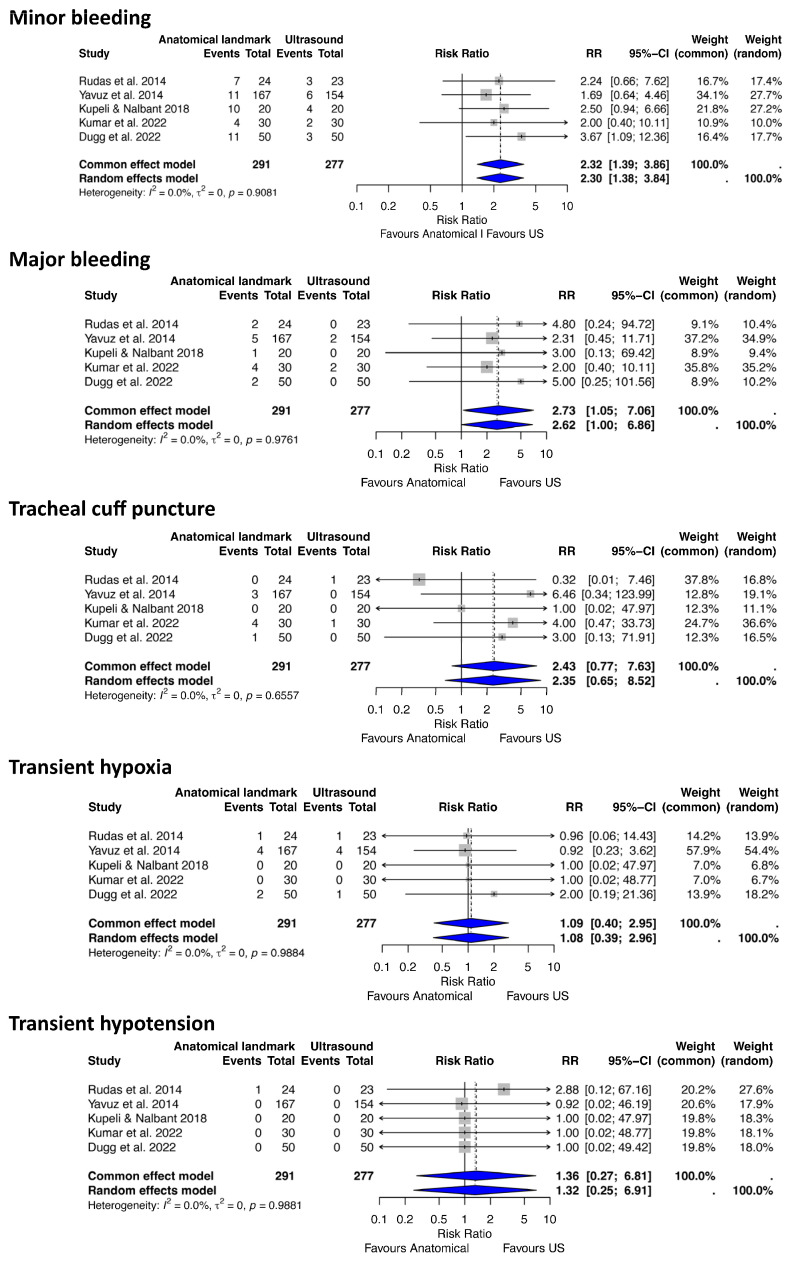
Forest plots demonstrating the effect of anatomical landmark versus ultrasound-guided techniques on minor and major bleeding, transient hypotension, transient hypoxia, and endotracheal tube cuff puncture after percutaneous tracheostomy. RR risk ratio, CI confidence interval [7,37,38,39,40].

For this analysis of complications, we included five RCT studies [7,37,40] which involved 568 patients who underwent PDT (291 ALG, 277 USG) (Figure 2).

Minor bleeding

Our analysis identified 61 minor bleeding events and showed a statistically significant increase in the risk of minor bleeding with the ALG approach compared to the USG approach (RR 2.30, 95% CI 1.38–3.84, *p* = 0.001, I^2^ = 0%, 95% CI 0–79.2%, Q = 1.01).

Major bleeding

Eighteen major bleeding events were recorded. The analysis showed a statistically significant difference between the two approaches, with clinically relevant results indicating a higher risk of major bleeding with the ALG approach compared to the USG approach (RR 2.62, 95% CI 1.00–6.86, *p* = 0.04, I^2^ = 0%, 95% CI 0–79.2%, Q = 0.47).

Transient hypoxia

The total number of transient hypoxia events was 13. The overall effect was not statistically significant between the two approaches (RR 1.07, 95% CI 0.39–2.96, *p* = 0.9, I^2^ = 0%, 95% CI 0–79.2%, Q = 0.32), suggesting a comparable clinically relevant risk of transient hypoxia.

Transient hypotension

One transient hypotension event was recorded. The overall random-effects model suggested there was no statistically significant difference in the risk of transient hypotension between the two approaches (RR 1.32, 95% CI 0.25–6.91, *p* = 0.74, I^2^ = 0.0%, 95% CI 0.0–79.2%, Q = 0.33).

Tracheal cuff puncture

Ten endotracheal cuff puncture events were recorded. The analysis showed no statistically significant difference in the risk of endotracheal tube cuff puncture between the ALG and USG approaches (RR 2.35, 95% CI 0.65–8.52, *p* = 0.19, I^2^ = 0%, 95% CI 0–79.2%, Q = 2.44), although the ALG approach was associated with approximately 2.4 times the risk of cuff-related complications compared to the USG approach.

Other notable complications during assisted PDT reported in these studies included two cases of paratracheal placement [38] and two cases of subcutaneous emphysema [40] with ALG, and one case each of paratracheal placement [38] and subcutaneous emphysema [40] with USG. Due to the low number of events, statistical analysis of these complications was not feasible.

(b) Ultrasound vs. Bronchoscopy-Guided PDT

**Figure 3:**
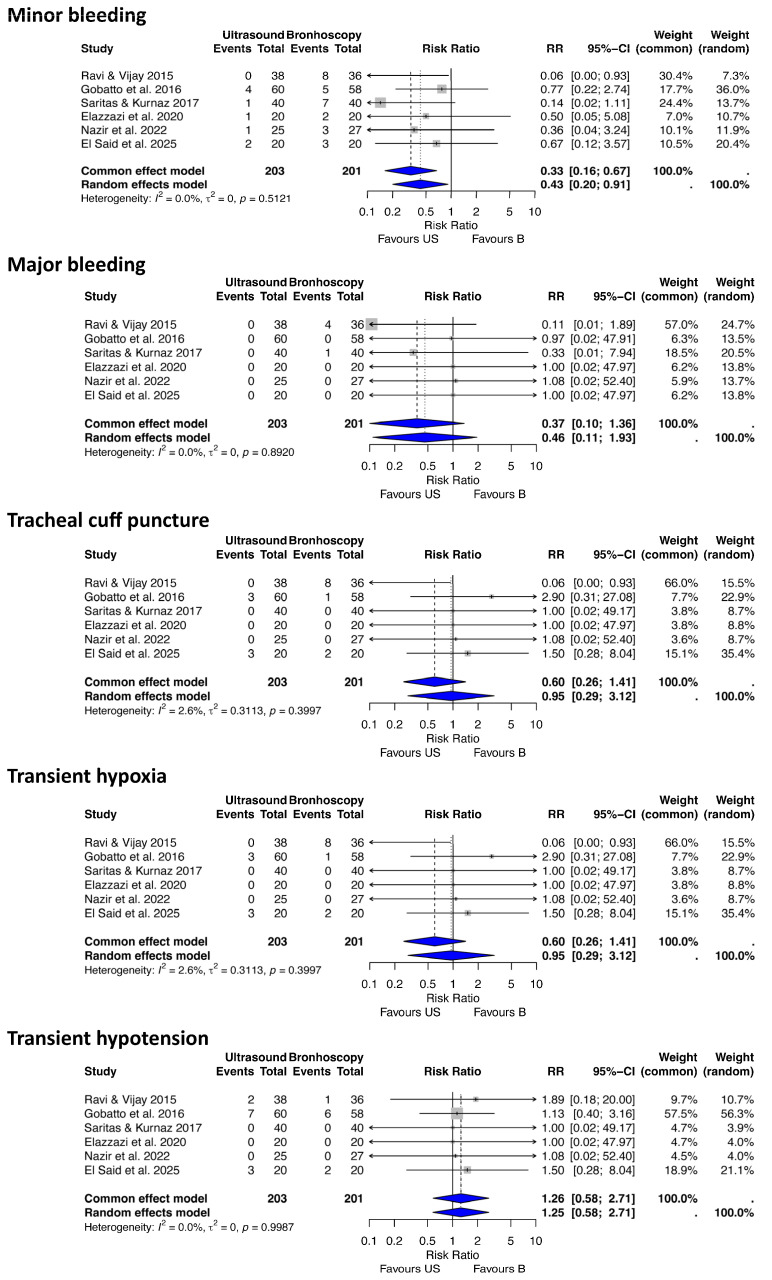
Forest plots demonstrating the effect of ultrasound versus bronchoscopy-guided techniques on minor and major bleeding, endotracheal tube cuff puncture, and transient hypoxia after percutaneous tracheostomy. RR risk ratio, CI confidence interval [31,41,42,43,44,45].

For this analysis of complications after PDT, we included six RCT studies [9,31,41,42,43,44] comparing different procedure-related complications between USG and BG PDT techniques, which involved 404 patients (203 USG, 201 BG) (Figure 3).

Minor bleeding

The analysis identified 37 minor bleeding events. The random-effects model suggested a 57% reduction in the risk of minor bleeding with USG compared to BG PDT (RR 0.42, 95% CI 0.2–0.91, *p* = 0.02, I^2^ = 0.0%, 95% CI 0–74.6%, Q = 4.26).

Major bleeding

Five major bleeding events were recorded. The analysis indicated a trend toward a reduced risk of major bleeding with USG compared to BG PDT, though this was not statistically significant (RR 0.46, 95% CI 0.10–1.93, *p* = 0.28, I^2^ = 0%, 95% CI 0–74.6%, Q = 1.68).

Transient hypoxia

Ten transient hypoxia events were recorded. The analysis showed no statistically significant difference in the risk of transient hypoxia between USG and BG PDT, although a reduced risk of hypoxia with USG PDT was suggested from a clinical perspective (RR 0.37, 95% CI 0.10–1.30, *p* = 0.11, I^2^ = 0%, 95% CI 0–74.6%, Q = 1.98).

Transient hypotension

Twenty-one transient hypotension events were recorded across the studies. The analysis showed no statistically significant difference in the risk of transient hypotension between USG and BG PDT (RR 1.25, 95% CI 0.58–2.71, *p* = 0.57, I^2^ = 0%, 95% CI 0–74.6%, Q = 0.23).

Tracheal cuff puncture

Seventeen endotracheal cuff puncture events were recorded. The pooled risk ratio (RR) for endotracheal cuff puncture indicated no statistically significant difference between USG and BG PDT (RR 0.95, 95% CI 0.29–3.12, *p* = 0.92, I^2^ = 2.6%, 95% CI 0–75.3%, Q = 5.13).

Other notable complications during assisted PDT reported in these studies included one case each of pneumothorax [31], subcutaneous emphysema [44], and procedure failure [31] with BG, and one case each of pneumothorax [42], mediastinitis [31], and procedure failure [31], along with two cases of paratracheal placement [42], with USG. Due to the low number of events, statistical analysis of these complications was not feasible.

(c) Anatomical landmark vs. Bronchoscopy-Guided Tracheostomy

**Figure 4:**
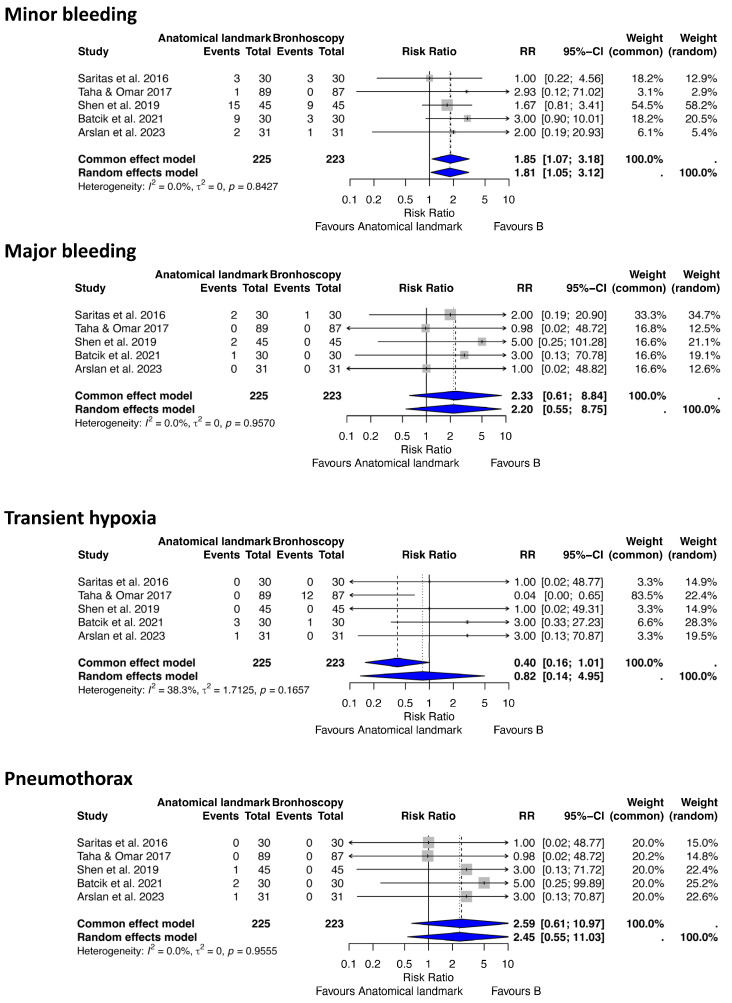
Forest plots demonstrating the effect of anatomical landmark versus bronchoscopy-guided techniques on minor and major bleeding, endotracheal tube cuff puncture, and pneumothorax after percutaneous tracheostomy. RR risk ratio, CI confidence interval [22,45,46,47,48].

For this analysis of complications after PDT, we included five RCT studies [22,45,46,47,48] comparing different procedure-related complications between ALG and BG PDT techniques, involving 448 patients (225 ALG, 223 BG) (Figure 4).

Minor bleeding

The analysis identified 46 minor bleeding events. The random-effects model demonstrated a significantly higher risk of minor bleeding with ALG compared to BG PDT (RR 1.81, 95% CI 1.05–3.12, *p* = 0.03, I^2^ = 0%, 95% CI 0–79.2%, Q = 1.41).

Major bleeding

Six major bleeding events were recorded. The analysis indicated a trend toward a reduced risk of major bleeding with BG compared to ALG PDT, though this was not statistically significant (RR 2.20, 95% CI 0.55–8.75, *p* = 0.26, I^2^ = 0%, 95% CI 0–79.2%, Q = 0.65).

Transient hypoxia

Seventeen transient hypoxia events were recorded. The analysis showed no statistically significant difference in the risk of transient hypoxia between ALG and BG PDT (RR 0.82, 95% CI 0.14–4.95, *p* = 0.82, I^2^ = 38.3%, 95% CI 0–77.1%, Q = 6.49).

Pneumothorax

Four pneumothorax events were recorded. The analysis suggested a higher clinical risk of pneumothorax with ALG compared to BG PDT, but no statistically significant difference was found (RR 2.45, 95% CI 0.55–11.03, *p* = 0.24, I^2^ = 0%, 95% CI 0–79.2%, Q = 0.67).

Other notable complications during assisted PDT reported in these studies included five endotracheal cuff punctures [45], two paratracheal placements [48], and three cases of posterior tracheal wall damage, one of which resulted in a tracheo-esophageal fistula [45,48]. Due to the low number of events, we were not able to undergo statistical analysis on this data. There were no transient hypotension, tracheal ring fractures, mediastinitis or procedure failure events recorded in these studies.

**Figure 5:**
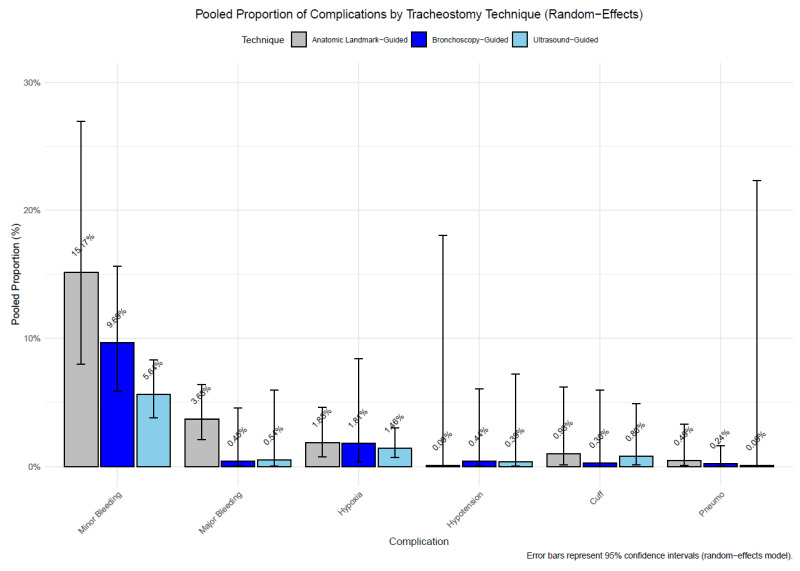
Pooled proportions of complications (minor bleeding, major bleeding, hypoxia, endotracheal tube cuff puncture, and hypotension) by technique (anatomical landmark-guided, ultrasound-guided, bronchoscopy-guided) in PDT. Bars represent random-effects model proportions, with error bars indicating 95% confidence intervals.

To better visualize the incidence of complications for each technique independently, we conducted a meta-analysis of pooled proportions of complications in PDT, using all studies listed in Table 1. A statistically significant difference was found for minor bleeding, with ALG PDT (15.44%) showing a higher proportion than USG PDT (5.52%, adjusted *p* = 0.00006). No other comparisons reached statistical significance after adjustment (adjusted *p* > 0.05), likely due to low event rates for transient hypoxia, transient hypotension, endotracheal tube cuff puncture, and pneumothorax complications (Figure 5).

Procedure Time

**Figure 6:**
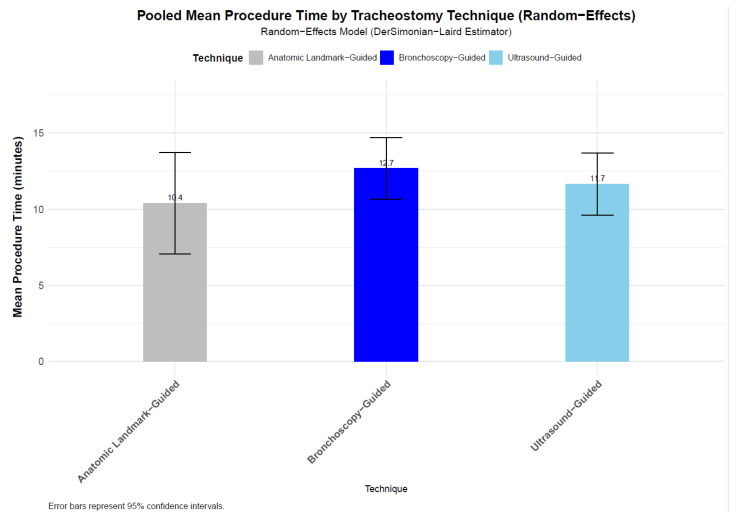
Bar chart of pooled mean procedure times with 95% confidence intervals for tracheostomy techniques, with no significant differences confirmed by pairwise and Wald-type tests. The pooled mean procedure times were 10.4 min (95% CI: 7.05–13.72, SE = 1.7) for ALG, 11.7 min (95% CI: 9.61–13.7, SE = 1.04) for USG, and 12.7 min (95% CI: 10.65–14.71, SE = 1.04) for BG PDT techniques.

To assess the mean procedure time across different PDT guidance methods, we pooled mean procedure time from nine studies for ALG [22,37,38,39,40,45,46,47,48] (n = 516), ten studies for USG [9,31,37,38,39,40,41,42,43,44] (n = 479), and eleven studies for BG [9,22,31,41,42,43,44,45,46,47,48] (n = 425). The random-effects model yielded pooled mean procedure times of 10.4 min (95% CI: 7.05–13.72, SE = 1.7) for ALG, 11.7 min (95% CI: 9.61–13.7, SE = 1.04) for USG, and 12.7 min (95% CI: 10.65–14.71, SE = 1.04) for BG PDT techniques (Figure 6). Standard errors, calculated from the 95% confidence intervals, were 1.70 min, 1.04 min, and 1.04 min, respectively, indicating greater variability in the ALG group compared to the image-guided techniques. Pairwise comparisons of mean durations showed no statistically significant differences. The difference between ALG and USG was −1.27 min (SE_diff = 1.995, z = −0.637, unadjusted *p* = 0.524, Bonferroni-adjusted *p* = 1.000), between ALG and BG was −2.29 min (SE_diff = 1.992, z = −1.152, unadjusted *p* = 0.249, Bonferroni-adjusted *p* = 0.748), and between USG and BG was −1.02 min (SE_diff = 1.47, z = −0.696, unadjusted *p* = 0.486, Bonferroni-adjusted *p* = 1.000). These results indicate that the observed differences in procedure durations are likely due to random variation. An overall Wald-type test for differences across all three techniques yielded a Wald statistic of 1.413 (degrees of freedom = 2, *p* = 0.493), further confirming no statistically significant difference in procedure durations among the groups. The analysis accounted for multiple comparisons using the Bonferroni correction, ensuring robustness against Type I errors.

No statistically significant differences in PDT procedure durations were found across the three guidance techniques.

### 3.4. Risk of Bias Assessment and Quality of Evidence

The randomization process and outcome measurement domains raised some concerns in certain studies due to unclear allocation concealment methods and lack of blinding of outcome assessors, respectively. There was a low risk of bias for deviations from intended interventions, missing outcome data, and selection of reported results. Risk of bias assessment results for the included studies are provided in the Supplementary Materials.

The quality of evidence was moderate for minor bleeding, but low or very low for the other outcomes. The GRADE approach results are found in the Supplementary Materials.

### 3.5. Heterogeneity and Publication Bias

Heterogeneity was assessed using the I^2^ statistic, Tau^2^, and Chi^2^ test for each meta-analyzed outcome, with results presented in the forest plots. The included outcomes exhibited negligible statistical heterogeneity (I^2^), with values of 0% or less than 10%, except for transient hypoxia in the ALG versus BG comparison, which showed moderate heterogeneity of 38.3% (95% CI 0–77.1%). This heterogeneity may be attributed to differences in operator experience or procedural protocols. The random-effects model was used to account for all the measured outcomes. Regarding publication bias, although no evidence was detected via funnel plot asymmetry for any outcome, this analysis was excluded because none of the meta-analyses included 10 or more studies, limiting the diagnostic accuracy of the tests.

## 4. Discussion

The main objective of this meta-analysis was to compare the safety and efficacy of PDT performed under USG, BG, and ALG techniques. Our findings suggest that USG PDT offers significant advantages in minimizing bleeding complications. Specifically, compared with the ALG, USG PDT significantly reduced both minor (RR 2.30, 95% CI 1.38–3.84, *p* = 0.001) and major bleeding (RR 2.62, 95% CI 1.00–6.86, *p* = 0.04). In comparison with BG, USG PDT was also associated with a statistically significant reduction in minor bleeding (RR 0.42, 95% CI 0.2–0.91, *p* = 0.02), and a clinically relevant trend toward reduced risk of major bleeding and transient hypoxia. BG PDT appears to offer some safety advantages over ALG in terms of bleeding incidence, with ALG associated with a significantly higher risk of minor bleeding (RR 1.81, 95% CI 1.05–3.12, *p* = 0.03), and a clinically important but not-significant trend toward higher major bleeding. The meta-analysis of pooled proportions provided further insight into the comparative safety of the three techniques. The significantly higher proportion of minor bleeding in ALG (15.44%) compared to USG (5.52%, adjusted *p* = 0.00006) reinforces the superiority of USG in minimizing bleeding complications.

The magnitude of risk reduction with USG PDT is clinically relevant because bleeding remains one of the most concerning complications that can occur during the procedure, potentially leading to airway obstruction or hemodynamic instability. The reduced risk of bleeding with ultrasound aligns with the theoretical advantages of real-time vascular visualization and avoidance of vascular puncture. These findings corroborate prior observational studies suggesting ultrasound improves procedural safety by enabling better anatomical localization [49,50]. Bronchoscopy, by providing direct visualization of the tracheal lumen, may lower the risk of vascular injuries compared to the blind ALG approach [16,51].

No statistically significant differences were observed for non-bleeding complications like transient hypoxia and hypotension between ALG and USG, or for transient hypoxia between ALG and BG. Rare complications encountered in ALG and USG, such as paratracheal placement and subcutaneous emphysema, are clinically important as they can increase mortality risk and warrant further study due to low event rates. Moreover, no significant differences were found between USG and BG for transient hypotension or endotracheal tube cuff puncture. The presence of rare complications, such as pneumothorax, paratracheal placement, mediastinitis, and procedure failure, in both USG and BG groups highlights the inherent risks of PDT.

Notably, the ALG approach exhibited trends toward higher risks for endotracheal tube cuff puncture and pneumothorax compared to USG and BG, and was linked to other relevant complications, such as paratracheal placement and posterior tracheal wall damage, including one case of tracheo-esophageal fistula [48]. These complications, though rare, highlight the potential for serious adverse events with the blind ALG approach, particularly in patients with challenging anatomy. It is worth noting that USG PDT does not protect against posterior tracheal wall injury.

Procedure duration was similar across all approaches, with no significant differences among ALG (10.4 min), USG (11.7 min), and BG (12.7 min) techniques. The slightly longer times for image-guided techniques (USG and BG) may reflect the additional setup and visualization steps required, but the lack of statistical significance suggests that these differences are not clinically meaningful.

From a clinical standpoint, although this study focused on periprocedural complications and duration, clinical practice involves multiple factors in selecting the optimal PDT technique for each patient, and it is based on operator experience, familiarity with ultrasound or bronchoscopy, and appropriate patient selection. For instance, while PDT is feasible in obese patients, it carries a higher risk of complications compared to normal-weight individuals [52]; thus, selecting the safest technique is crucial [53]. Depending on the healthcare setting in which the PDT is performed, USG PDT appears to offer the safest profile, with BG PDT as a valid alternative. Hybrid techniques could offer the advantages of both approaches.

Prior meta-analyses, like Iftikhar et al. [54], reported comparable complication rates across techniques, but did not offer more of an in-depth analysis of complications, potentially underestimating clinically significant differences, whereas our study demonstrates USG’s benefit over ALG. In comparison to a meta-analysis by Gobbato et al. [55], which combined the number of minor and major complications and assessed outcomes as a composite, our analysis evaluated each individually. Lin et al. [56] found no significant major bleeding reduction with USG vs. ALG PDT, which was contradicted by Wen et al. [57]; our results reinforce the latter by focusing exclusively on RCTs.

In contrast to the mentioned meta-analyses, our study’s strengths include a rigorous methodology and comprehensive analysis, extending the literature search to include all available RCTs up to 2025 and assessing a broader range of procedure-related outcomes.

## 5. Limitations

Several limitations should be considered when interpreting these results. First, the small number of studies [5,6] and the low event rates for certain complications (major bleeding, transient hypotension, pneumothorax) limited the statistical power to detect differences, potentially leading to type II errors. Second, the included studies varied in patient populations, operator expertise, and procedural protocols, which may have introduced some heterogeneity, particularly for transient hypoxia in the ALG vs. BG comparison (I^2^ = 38.3%). Third, rare complications, such as paratracheal placement, pneumothorax, and mediastinitis, could not be analyzed statistically due to insufficient event rates, limiting our ability to draw definitive conclusions about these outcomes. Notably, isolated reports of severe events—including tracheoesophageal fistula and mediastinitis—emphasize the need for vigilance regardless of technique. Finally, the analysis did not account for long-term outcomes due to scarcity, and the limited trials per group preclude firm conclusions on rare adverse events. Additionally, risk of bias in certain domains and the low quality of evidence may affect reliability.

Despite these limitations, the findings carry important clinical implications. USG appears to provide a safer profile with respect to minor and major bleeding and may be considered a preferable approach where expertise and equipment are available. BG, while not associated with statistically superior outcomes in this analysis, remains valuable for real-time airway visualization and confirmation of cannula placement. The ALG, although feasible, was consistently associated with higher rates of bleeding and other complications with potentially greater variability in performance.

More research is required, and it should focus on larger, multicenter RCTs to evaluate rare but clinically significant complications, as well as cost-effectiveness analyses to adapt practice guideline recommendations, training, and suggestion of a standard procedure in the ICU. In addition, studies exploring hybrid approaches with synergistic benefits (ultrasound for vascular mapping and bronchoscopy for airway guidance) may further optimize the balance between safety and practicality.

## 6. Conclusions

USG PDT is associated with a significantly lower risk of minor and major bleeding compared to ALG and a lower risk of minor bleeding compared to BG. While no significant differences were found in non-bleeding complications or procedure times, the trends observed suggest potential clinical benefits of image-guided techniques, particularly USG. These findings support the use of USG as the preferred method for PDT in settings where ultrasound is available, with BG as a viable alternative to ALG in reducing bleeding and serious complications. Hybrid approaches using USG and BG could further improve the safety profile of PDT. Clinicians should weigh these benefits against practical considerations, such as equipment availability and operator training, when selecting a PDT guidance technique.

## Figures and Tables

**Table 1:** Overview of the RCT studies included in the meta-analysis.

	First Author (Year)	Design	Population	Intervention	Comparator	Outcome	Study Period and Country	Sample Size	Conclusion	Quality Score
Anatomical Landmark vs. Ultrasound-Guided Percutaneous Tracheostomy										
1	Kumar (2022) [38]	RCT	Critically ill patients aged >20 years requiring tracheostomy after prolonged mechanical ventilation	USG	ALG	Deviation from midline, number of passes/trials, duration of procedure, immediate peri-procedural complications	April 2019 to December 2020, India	60 (30 each group)	Ultrasound-guided PDT showed superiority over landmark PDT in terms of less number of trials, midline puncture and fewer complications. However, it took a little longer to perform USG-guided PDT.	Some concerns (primarily lack of reported allocation concealment and unblinded outcome assessment for some measures).
2	Dugg (2022) [39]	RCT	Critically ill ICU patients aged ≥18 years on prolonged mechanical ventilation requiring tracheostomy	USG	ALG	Efficiency (assessment time, procedure time), efficacy (number of attempts, complications), accuracy (deviation from midline, puncture site)	2020–2021 (approximate based on submission dates), India	100 (50 each group)	Ultrasound-guided PDT is associated with reduction in periprocedural complications as compared to landmark technique, although it takes slightly longer time.	Some concerns (mainly concealment not detailed; partial blinding may affect some outcomes, though primary outcome measurement was fairly objective).
3	Kupeli (2018) [40]	RCT	Critically ill adults, mean age 68, mean APACHE II 27.4	USG	ALG	Puncture success, complications	December 2017, Turkey	40 (20 each group)	Out-of-plane US had higher first-entry success, fewer complications	Some concerns RCT with randomization and complete follow-up; minor limitation from unspecified allocation concealment.
4	Rudas (2014) [7]	RCT	Long-term ventilated ICU adults	USG	ALG	Puncture accuracy, complications	March–December 2011, Australia	47 (23 US, 24 ALG)	Higher midline accuracy in US (87% vs. 50%, *p* = 0.006), fewer complications	Low RoB RCT with blinded assessors and complete follow-up; allocation concealment not detailed.
5	Yavuz (2014) [37]	RCT	Critically ill adults	USG	ALG	Complications, procedure time	December 2007–January 2011, Turkey	341 (166 US, 175 ALG)	Lower complications in US (7.8% vs. 15.0%, *p* = 0.054), longer time in US (*p* = 0.001)	Some concerns Randomization, institutional review board approval, complete follow-up, though blinding and allocation concealment details are not specified.
Ultrasound vs. Bronchoscopy-Guided Percutaneous Tracheostomy										
1	Gobatto (2016) [31]	RCT (Non-inferiority)	Critically ill, mechanically ventilated adults, mean age 48.4, 68.6% male	USG	BG	Procedure failure (1.7% both groups, 90% CI −5.57 to 5.85)	March 2014–May 2015, Brazil	118 (60 US, 58 B)	Non-inferiority met, minor complications 33.3% vs. 20.7% (*p* = 0.122)	Low RoB RCT with clear randomization, complete follow-up, and defined non-inferiority margin; minor limitation due to lack of blinding.
2	Sarıtas (2019) [9]	RCT	Critically ill adults, mean APACHE II 17.9	USG	BG	Hemorrhage, procedure duration	February–March 2017, Turkey	80 (40 US, 40 B)	Lower hemorrhage in US-PDT (*p* < 0.05), shorter duration in US-PDT (*p* < 0.05)	Low RoB RCT with randomization and complete data; lack of blinding of participants is a minor issue.
3	Ravi (2015) [41]	RCT	Critically ill obese adults	USG	BG	Complications, procedure time	February 2014–January 2015, India	74 (38 US, 36 B)	Lower complication rate in USPCT (32.1% vs. 75%, *p* < 0.05), shorter time	Some concerns RCT with randomization and complete data; lack of blinding is a minor limitation.
4	Elazzazi (2020) [42]	RCT	Critically ill patients in ICU with factors increasing procedural difficulty (e.g., morbid obesity, difficult anatomy, cervical spine precautions)	USG	BG	Value of US in assisting PDT (e.g., identifying cervical anatomy, vasculature, thyroid; preventing vascular puncture or other complications)	Not specified, Egypt (Ain Shams University)	40 (20/20)	Ultrasound has emerged as a potentially useful tool in assisting PDT, especially when factors increase technical difficulty; several studies demonstrate its value, but no specific comparative results provided in excerpt.	Some concerns (abstract-only; lacks full methods, results, or sample details)
5	Nazir (2022) [43]	RCT	Obese ICU patients requiring PDT	USG	BG	Procedure efficacy and postoperative complications (e.g., intra-procedural complications like bleeding, hypoxemia; operation time; no postoperative complications noted)	April 2020 to April 2021, Pakistan (Services Hospital, Lahore)	52 (25 US, 27 B)	Ultrasound-guided procedure is superior to bronchoscopy-guided PDT among obese ICU patients with a low percentage of intra- and post-operative complications; shorter operation time in ultrasound group.	Some concerns (RCT with randomization and clear outcomes)
6	El Said (2025) [44]	RCT	Mechanically ventilated critically ill patients in ICU requiring prolonged ventilation	USG	BG	Safety and effectiveness (e.g., procedural duration, complications, outcomes like P/F ratio, ICU/hospital stay, mortality; no significant differences except shorter duration in ultrasound)	Not specified, Egypt (Menoufia University Hospital)	40 (20 US, 20 B)	Ultrasound- or bronchoscopy-guided PDT showed comparable results in terms of complications and outcomes in critically ill patients; however, a significant difference was noted in procedural duration (shorter with ultrasound).	Low RoB (RCT with randomization, ethical approval, and CONSORT compliance)
	Anatomical Landmark vs. Bronchoscopy-Guided Percutaneous Tracheostomy									
1	Saritas (2016) [45]	RCT	Critically ill ventilated ICU patients	ALG	BG	Complications, number of needle passes, procedure duration	2013–2014, Turkey	60 (30 vs. 30)	FOB significantly reduced complications and needle passes; longer procedure time	Low RoB (well-described RCT, prospective, balanced groups)
2	Taha (2017) [22]	RCT	Critically ill adults, mean age 55.6	ALG	BG	Procedure time, complications	January–May 2017, UAE	176 (89 AL, 87 B)	Procedure time 5 vs. 12 min, no major complications in either group	Moderate RoB (randomization simple, but some methodological limitations)
3	Shen (2019) [46]	RCT	Critically ill adults, mean SOFA 7.5	ALG	BG	First-time success, complications	May–December 2018, China	90 (45 each group)	Higher first-time success in FOB-PDT (93.3% vs. 64.4%, *p* < 0.05)	Low RoB RCT with randomization and complete data; minor limitation due to lack of blinding.
4	Batcik (2021) [47]	RCT	ICU patients with prolonged ventilation	ALG	BG	Procedure time, blood gases, complications, ICU stay	Not specified (study ~2020–2021), Turkey	60 (30 vs. 30)	FOB prolonged procedure time; complication rates similar	Some concerns (small sample, randomization but limited outcome detail)
5	Arslan (2023) [48]	RCT	ICU patients on ventilation	ALG	BG	Complications, mortality, procedure time, ICU stay	2022–2023, Turkey	62 (31 vs. 31)	FOB reduced complications, ICU stay, and procedure time; no mortality difference	Low RoB (prospective, randomized, clear reporting)

RCT—randomized controlled trial, USG—ultrasound guided, ALG—anatomic landmark guided, BG—bronchoscopy guided, ICU—intensive care unit, RoB—risk of bias.

## Data Availability

All data are available, either analyzed as figures or tables presented in the current manuscript or as raw data upon request by any external collaborator or reviewer.

## Supplementary Materials

The following supporting information can be downloaded at https://www.mdpi.com/article/10.3390/jcm14228050/s1. Figure S1. The risk of bias assessment at study and at domain level for the ALG vs USG studies (for outcomes minor and major bleeding, transient hypotension, transient hypoxia, and endotracheal tube cuff puncture); Figure S2. The risk of bias assessment at study and at domain level for the USG vs BG studies (studies (for outcomes minor and major bleeding, transient hypotension, transient hypoxia, and endotracheal tube cuff puncture); Figure S3. The risk of bias assessment at study and at domain level for the ALG vs BG studies (for outcomes minor and major bleeding, endotracheal tube cuff puncture, and pneumothorax); Table S1. PRISMA checklist; Table S2. The detailed search key; Table S3. Summaryof findings table of the quality of evidence for theminor bleeding, major bleeding, transient hypoxia, hypotension, endotracheal tube cuff puncture, and pneumothorax.
