# Supplementary material for: Complications of Percutaneous Tracheostomy-Assisting Techniques in Critically Ill Patients: A Systematic Review and Meta-Analysis of Randomized Controlled Trials
Source: J Clin Med. 2025 Nov 13;14(22):8050. doi: 10.3390/jcm14228050 (PMC12653206; doi:10.3390/jcm14228050)
Supplement: Supplementary file 1 [file jcm-14-08050-s001.zip › jcm-3963620-supplementary.pdf]

SUPPLEMENTARY MATERIAL

TITLE

Complications of Percutaneous Tracheostomy-Assisting Techniques in Critically Ill Patients: A Systematic Review and Meta-Analysis of Randomized Controlled Trials

AUTHORS

Olga Grajdieru [1,2], Constantin Bodolea [1,3], Vlad Moisoiu [4], Cristina Petrișor [1,2], Catalin Constantinescu [1,2,4,*]

AFFILIATIONS

[1] Department of Anesthesia and Intensive Care, Iuliu Hatieganu University of Medicine and Pharmacy, 400012 Cluj-Napoca, Romania; olgadoinag@yahoo.com (O.G.); constantin.bodolea@umfcluj.ro (C.B.); petrisor.cristina@umfcluj.ro (C.P.)
[2] Intensive Care Unit, Emergency Clinical Hospital, 400347 Cluj-Napoca, Romania
[3] Municipal Clinical Hospital, 400139 Cluj-Napoca, Romania
[4] MedFuture Research Center for Advanced Medicine, Iuliu Hatieganu University of Medicine and Pharmacy, 400349 Cluj-Napoca, Romania; vlad.moisoiu@gmail.com

* Correspondence: constantinescu.catalin@umfcluj.ro

Figure Legends

Figure S1. The risk of bias assessment at study and at domain level for the ALG vs USG studies (for outcomes minor and major bleeding, transient hypotension, transient hypoxia, and endotracheal tube cuff puncture).

Figure S2. The risk of bias assessment at study and at domain level for the USG vs BG studies (studies (for outcomes minor and major bleeding, transient hypotension, transient hypoxia, and endotracheal tube cuff puncture).

Figure S3. The risk of bias assessment at study and at domain level for the ALG vs BG studies (for outcomes minor and major bleeding, endotracheal tube cuff puncture, and pneumothorax).

ALG – anatomic landmark guided; USG – ultrasound guided; BG – bronchoscopy guided

Table Legends

Table S1. PRISMA checklist.

Table S2. The detailed search key.

Table S3. Summary of findings table of the quality of evidence for the minor bleeding, major bleeding, transient hypoxia, hypotension, endotracheal tube cuff puncture, and pneumothorax.

Table S1. PRISMA checklist

Section and Topic	Item #	Checklist item	Location where item is reported (PAGE)
**TITLE**			
Title	1	Identify the report as a systematic review.	1
**ABSTRACT**			
Abstract	2	See the PRISMA 2020 for Abstracts checklist.	2
**INTRODUCTION**			
Rationale	3	Describe the rationale for the review in the context of existing knowledge.	2-3
Objectives	4	Provide an explicit statement of the objective(s) or question(s) the review addresses.	2-3
**METHODS**			
Eligibility criteria	5	Specify the inclusion and exclusion criteria for the review and how studies were grouped for the syntheses.	3
Information sources	6	Specify all databases, registers, websites, organisations, reference lists and other sources searched or consulted to identify studies. Specify the date when each source was last searched or consulted.	4
Search strategy	7	Present the full search strategies for all databases, registers and websites, including any filters and limits used.	4 and Supplementary Table S2
Selection process	8	Specify the methods used to decide whether a study met the inclusion criteria of the review, including how many reviewers screened each record and each report retrieved, whether they worked independently, and if applicable, details of automation tools used in the process.	4
Data collection process	9	Specify the methods used to collect data from reports, including how many reviewers collected data from each report, whether they worked independently, any processes for obtaining or confirming data from study investigators, and if applicable, details of automation tools used in the process.	4
Data items	10a	List and define all outcomes for which data were sought. Specify whether all results that were compatible with each outcome domain in each study were sought (e.g. for all measures, time points, analyses), and if not, the methods used to decide which results to collect.	5
	10b	List and define all other variables for which data were sought (e.g. participant and intervention characteristics, funding sources). Describe any assumptions made about any missing or unclear information.	5
Study risk of bias assessment	11	Specify the methods used to assess risk of bias in the included studies, including details of the tool(s) used, how many reviewers assessed each study and whether they worked independently, and if applicable, details of automation tools used in the process.	5
Effect measures	12	Specify for each outcome the effect measure(s) (e.g. risk ratio, mean difference) used in the synthesis or presentation of results.	6-7
Synthesis methods	13a	Describe the processes used to decide which studies were eligible for each synthesis (e.g. tabulating the study intervention characteristics and comparing against the planned groups for each synthesis (item #5)).	6-7
	13b	Describe any methods required to prepare the data for presentation or synthesis, such as handling of missing summary statistics, or data conversions.	6-7

Section and Topic	Item #	Checklist item	Location where item is reported (PAGE)
	13c	Describe any methods used to tabulate or visually display results of individual studies and syntheses.	6-7
	13d	Describe any methods used to synthesize results and provide a rationale for the choice(s). If meta-analysis was performed, describe the model(s), method(s) to identify the presence and extent of statistical heterogeneity, and software package(s) used.	6-7
	13e	Describe any methods used to explore possible causes of heterogeneity among study results (e.g. subgroup analysis, meta-regression).	6-7
	13f	Describe any sensitivity analyses conducted to assess robustness of the synthesized results.	6-7
Reporting bias assessment	14	Describe any methods used to assess risk of bias due to missing results in a synthesis (arising from reporting biases).	6-7
Certainty assessment	15	Describe any methods used to assess certainty (or confidence) in the body of evidence for an outcome.	5
**RESULTS**			
Study selection	16a	Describe the results of the search and selection process, from the number of records identified in the search to the number of studies included in the review, ideally using a flow diagram.	8 (Figure 1)
	16b	Cite studies that might appear to meet the inclusion criteria, but which were excluded, and explain why they were excluded.	-
Study characteristics	17	Cite each included study and present its characteristics.	8-10 and Table 1
Risk of bias in studies	18	Present assessments of risk of bias for each included study.	Table 1 and Supplementary Figures S1-S3
Results of individual studies	19	For all outcomes, present, for each study: (a) summary statistics for each group (where appropriate) and (b) an effect estimate and its precision (e.g. confidence/credible interval), ideally using structured tables or plots.	Figures 2-5
Results of syntheses	20a	For each synthesis, briefly summarise the characteristics and risk of bias among contributing studies.	Table 1 and Supplementary Figures S1-S3
	20b	Present results of all statistical syntheses conducted. If meta-analysis was done, present for each the summary estimate and its precision (e.g. confidence/credible interval) and measures of statistical heterogeneity. If comparing groups, describe the direction of the effect.	Figures 2-5
	20c	Present results of all investigations of possible causes of heterogeneity among study results.	-
	20d	Present results of all sensitivity analyses conducted to assess the robustness of the synthesized results.	3, 5, 6

Section and Topic	Item #	Checklist item	Location where item is reported (PAGE)
Reporting biases	21	Present assessments of risk of bias due to missing results (arising from reporting biases) for each synthesis assessed.	-
Certainty of evidence	22	Present assessments of certainty (or confidence) in the body of evidence for each outcome assessed.	Supplementary Table S3
**DISCUSSION**			
Discussion	23a	Provide a general interpretation of the results in the context of other evidence.	17
	23b	Discuss any limitations of the evidence included in the review.	18
	23c	Discuss any limitations of the review processes used.	18
	23d	Discuss implications of the results for practice, policy, and future research.	19
**OTHER INFORMATION**			
Registration and protocol	24a	Provide registration information for the review, including register name and registration number, or state that the review was not registered.	3
	24b	Indicate where the review protocol can be accessed, or state that a protocol was not prepared.	3
	24c	Describe and explain any amendments to information provided at registration or in the protocol.	3
Support	25	Describe sources of financial or non-financial support for the review, and the role of the funders or sponsors in the review.	19
Competing interests	26	Declare any competing interests of review authors.	20
Availability of data, code and other materials	27	Report which of the following are publicly available and where they can be found: template data collection forms; data extracted from included studies; data used for all analyses; analytic code; any other materials used in the review.	20

Table S2. The detailed search key

Database	Search Key
**CENTRAL**	("Percutaneous Tracheostomy" OR "Percutaneous Tracheotomy" OR "Percutaneous Dilatational Tracheostomy") AND ("Intensive Care Units" OR "Critical Care" OR "ICU") AND ("Bronchoscopy" OR "Ultrasonography" OR "Ultrasound Guidance" OR "Endoscopic Guidance" OR "Assisting Techniques" OR "Guided") AND ("Safety" OR "Complications" OR "Mortality" OR "Procedure Duration" OR "Success Rate")
**PubMed**	("Percutaneous Tracheostomy"[Mesh] OR "Percutaneous Tracheotomy" OR "Percutaneous Dilatational Tracheostomy") AND ("Intensive Care Units"[Mesh] OR "Critical Care" OR "ICU") AND ("Bronchoscopy"[Mesh] OR "Ultrasonography" OR "Ultrasound Guidance" OR "Endoscopic Guidance" OR "Assisting Techniques" OR "Guided") AND ("Safety" OR "Complications" OR "Mortality" OR "Procedure Duration" OR "Success Rate")
**Scopus**	(("Percutaneous Tracheostomy" OR "Percutaneous Tracheotomy" OR "Percutaneous Dilatational Tracheostomy") AND ("Intensive Care Units" OR "Critical Care" OR "ICU") AND ("Bronchoscopy" OR "Ultrasonography" OR "Ultrasound Guidance" OR "Endoscopic Guidance" OR "Assisting Techniques" OR "Guided") AND ("Safety" OR "Complications" OR "Mortality" OR "Procedure Duration" OR "Success Rate"))
**Web of Science**	TS=("Percutaneous Tracheostomy" OR "Percutaneous Tracheotomy" OR "Percutaneous Dilatational Tracheostomy") AND TS=("Intensive Care Units" OR "Critical Care" OR "ICU") AND TS=("Bronchoscopy" OR "Ultrasonography" OR "Ultrasound Guidance" OR "Endoscopic Guidance" OR "Assisting Techniques" OR "Guided") AND TS=("Safety" OR "Complications" OR "Mortality" OR "Procedure Duration" OR "Success Rate")

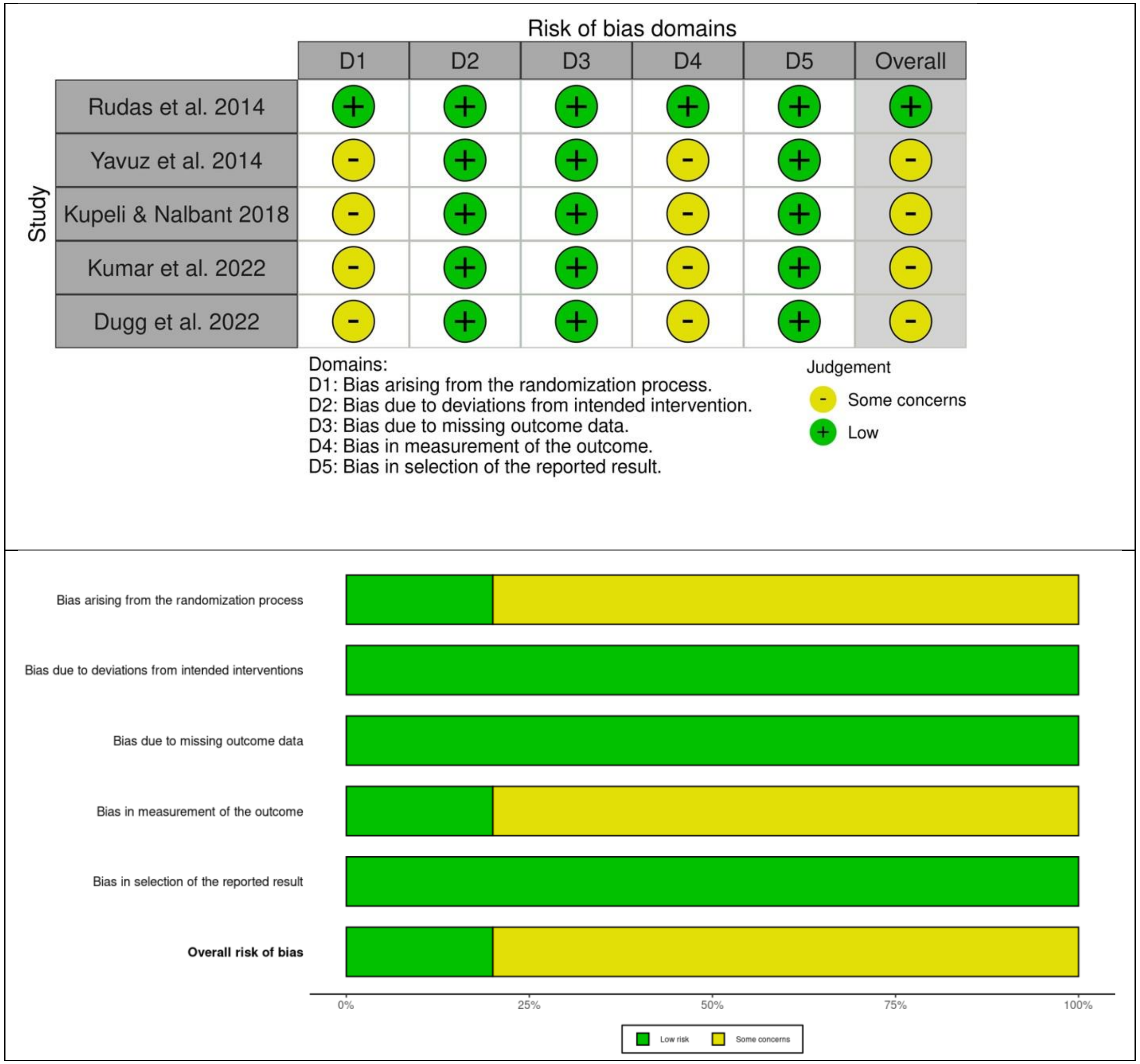

Figure S1. The risk of bias assessment at study and at domain level for the ALG vs USG studies (for outcomes minor and major bleeding, transient hypotension, transient hypoxia, and endotracheal tube cuff puncture).

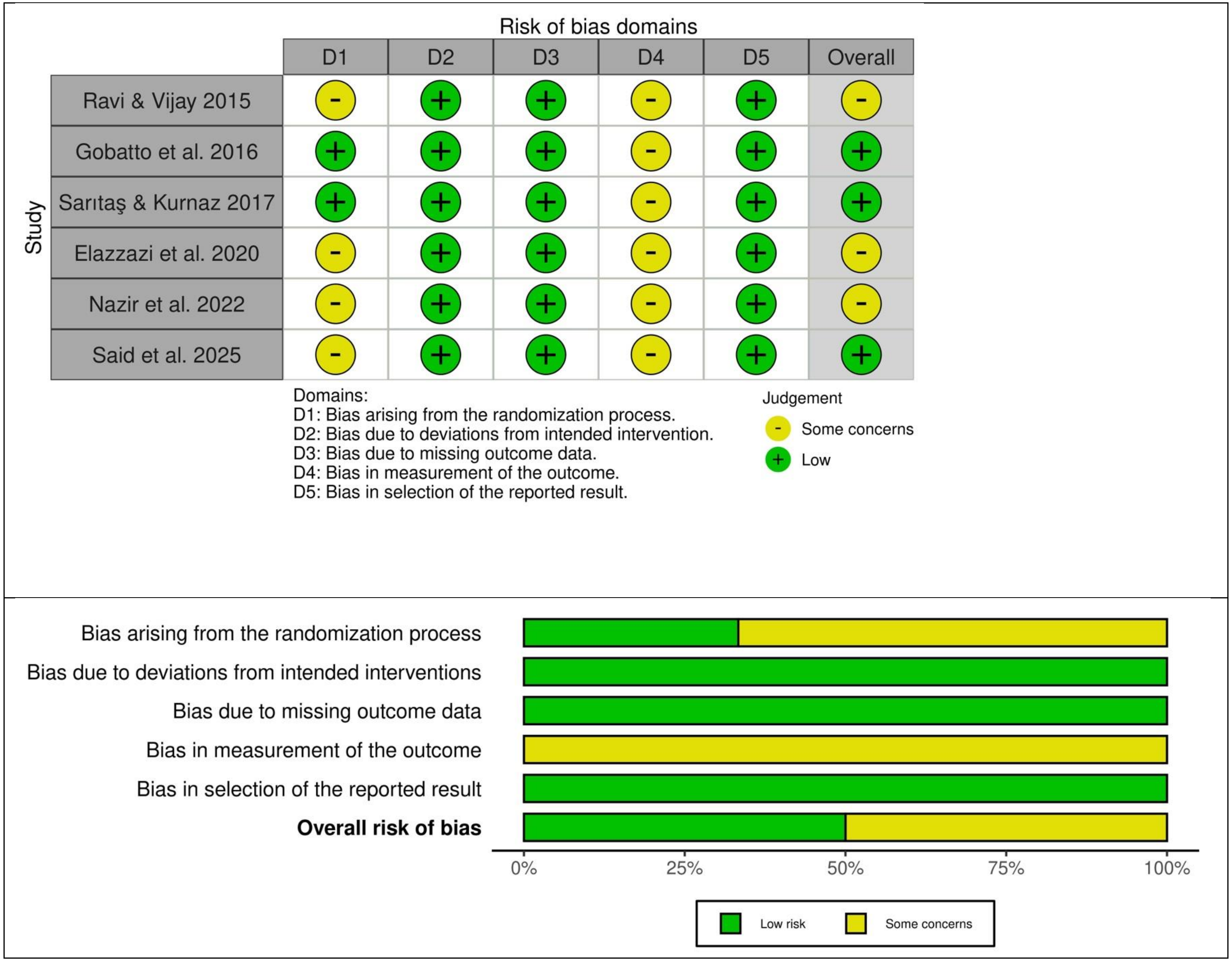

Figure S2. The risk of bias assessment at study and at domain level for the USG vs BG studies (studies (for outcomes minor and major bleeding, transient hypotension, transient hypoxia, and endotracheal tube cuff puncture).

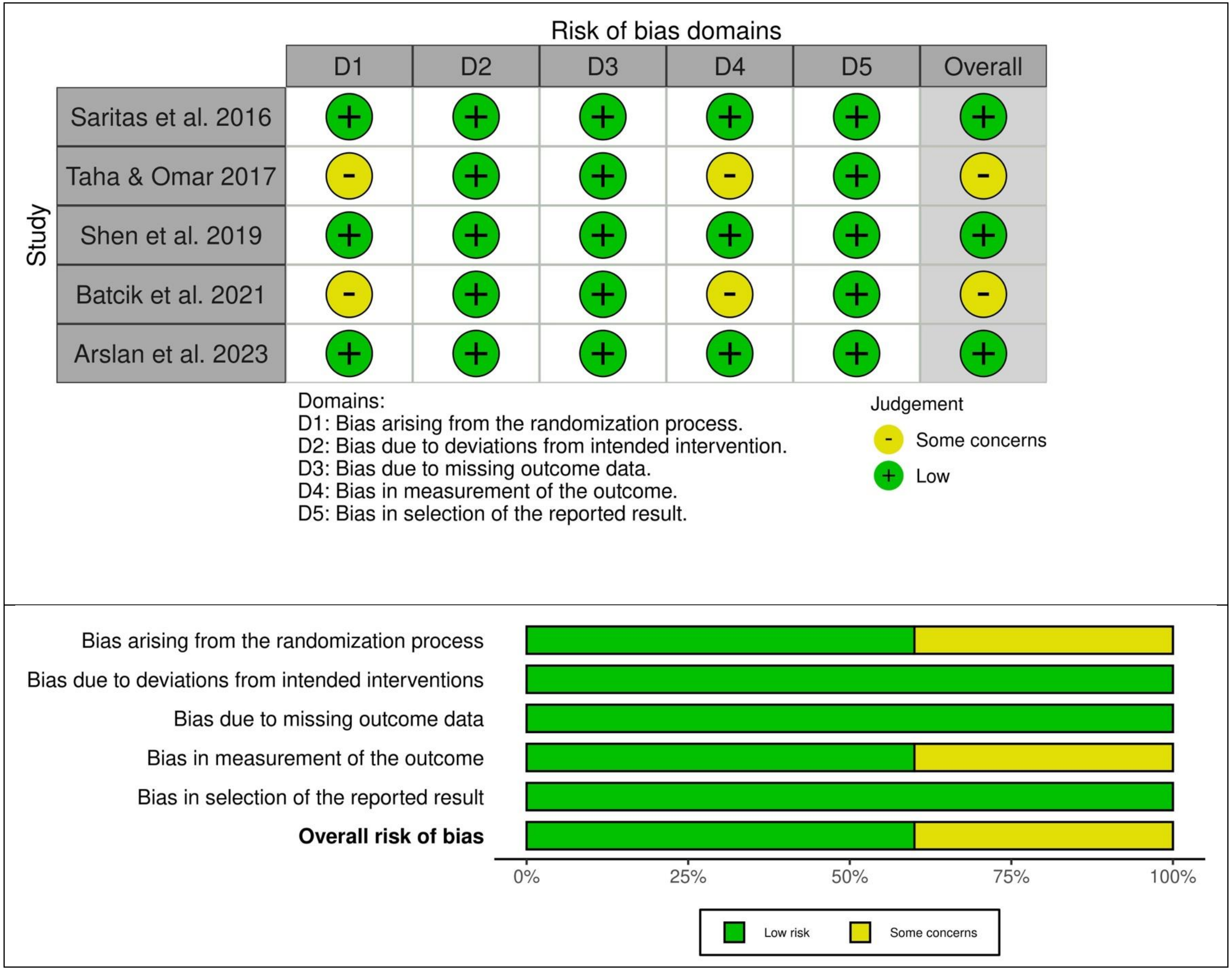

Figure S3. The risk of bias assessment at study and at domain level for the ALG vs BG studies (for outcomes minor and major bleeding, endotracheal tube cuff puncture, and pneumothorax).

Table S3. Summary of findings table of the quality of evidence for the minor bleeding, major bleeding, transient hypoxia, hypotension, endotracheal tube cuff puncture, and pneumothorax.

Outcome	№ of studies	Study design	Risk of bias	Inconsistency	Indirectness	Imprecision	Other considerations	№ of patients (ALG/USG/BG)	Effect (Relative, 95% CI)	Effect (Absolute, 95% CI)	Certainty	Importance
**Minor Bleeding (ALG vs. USG)**	5	Randomized trials	Serious[a]	Not serious[b]	Not serious[c]	Not serious[d]	None	291/277/-	RR 2.30 (1.38–3.84)	85 more per 1,000 (from 25 more to 185 more)[a*]	⊕⊕⊕◯ Moderate	CRITICAL
**Minor Bleeding (USG vs. BG)**	6	Randomized trials	Serious[a]	Not serious[b]	Not serious[c]	Not serious[d]	None	-/203/201	RR 0.42 (0.20–0.91)	81 fewer per 1,000 (from 111 fewer to 13 fewer)[b*]	⊕⊕⊕◯ Moderate	CRITICAL
**Minor Bleeding (ALG vs. BG)**	5	Randomized trials	Serious[a]	Not serious[b]	Not serious[c]	Not serious[d]	None	225/-/223	RR 1.81 (1.05–3.12)	58 more per 1,000 (from 4 more to 152 more)[c*]	⊕⊕⊕◯ Moderate	CRITICAL
**Major Bleeding (ALG vs. USG)**	5	Randomized trials	Serious[a]	Not serious[b]	Not serious[c]	Serious[d]	None	291/277/-	RR 2.62 (1.00–6.86)	23 more per 1,000 (from 0 more to 84 more)[d*]	⊕⊕◯◯ Low	CRITICAL
**Major Bleeding (USG vs. BG)**	6	Randomized trials	Serious[a]	Not serious[b]	Not serious[c]	Serious[d]	None	-/203/201	RR 0.46 (0.10–1.93)	13 fewer per 1,000 (from 22 fewer to 23 more)[e*]	⊕⊕◯◯ Low	CRITICAL
**Major Bleeding (ALG vs. BG)**	5	Randomized trials	Serious[a]	Not serious[b]	Not serious[c]	Serious[d]	None	225/-/223	RR 2.20 (0.55–8.75)	5 more per 1,000 (from 2 fewer to 35 more)[f*]	⊕⊕◯◯ Low	CRITICAL
**Transient Hypoxia (ALG vs. USG)**	5	Randomized trials	Serious[a]	Not serious[b]	Not serious[c]	Serious[d]	None	291/277/-	RR 1.07 (0.39–2.96)	2 more per 1,000 (from 13 fewer to 43 more)[g*]	⊕⊕◯◯ Low	CRITICAL
**Transient Hypoxia (USG vs. BG)**	6	Randomized trials	Serious[a]	Not serious[b]	Not serious[c]	Serious[d]	None	-/203/201	RR 0.37 (0.10–1.30)	28 fewer per 1,000 (from 40 fewer to 13 more)[h*]	⊕⊕◯◯ Low	CRITICAL
**Transient Hypoxia (ALG vs. BG)**	5	Randomized trials	Serious[a]	Serious[b]	Not serious[c]	Serious[d]	None	225/-/223	RR 0.82 (0.14–4.95)	10 fewer per 1,000 (from 50 fewer to 230 more)[i*]	⊕◯◯◯ Very low	CRITICAL
**Transient Hypotension (ALG vs. USG)**	5	Randomized trials	Serious[a]	Not serious[b]	Not serious[c]	Serious[d]	None	291/277/-	RR 1.32 (0.25–6.91)	1 more per 1,000 (from 1 fewer to 11 more)[j*]	⊕⊕◯◯ Low	CRITICAL
**Transient Hypotension (USG vs. BG)**	6	Randomized trials	Serious[a]	Not serious[b]	Not serious[c]	Serious[d]	None	-/203/201	RR 1.25 (0.58–2.71)	11 more per 1,000 (from 19 fewer to 77 more)[k*]	⊕⊕◯◯ Low	CRITICAL
**Endotracheal Cuff Puncture (ALG vs. USG)**	5	Randomized trials	Serious[a]	Not serious[b]	Not serious[c]	Serious[d]	None	291/277/-	RR 2.35 (0.65–8.52)	10 more per 1,000 (from 3 fewer to 54 more)[l*]	⊕⊕◯◯ Low	CRITICAL
**Endotracheal Cuff Puncture (USG vs. BG)**	6	Randomized trials	Serious[a]	Not serious[b]	Not serious[c]	Serious[d]	None	-/203/201	RR 0.95 (0.29–3.12)	3 fewer per 1,000 (from 39 fewer to 116 more)[m*]	⊕⊕◯◯ Low	CRITICAL
**Pneumothorax (ALG vs. BG)**	5	Randomized trials	Serious[a]	Not serious[b]	Not serious[c]	Serious[d]	None	225/-/223	RR 2.45 (0.55–11.03)	3 more per 1,000 (from 1 fewer to 22 more)[n*]	⊕⊕◯◯ Low	CRITICAL

ALG – anatomic- landmark guided; USG – ultrasound-guided; BG – bronchoscopy-guided; CI: confidence interval; RR: risk ratio

Footnotes:

[a] There were some concerns about the risk of bias in most of the studies included for the analysis of this outcome due to unclear allocation concealment in the randomization process and lack of blinding of assessors for the outcome measurement. Otherwise, there were no significant differences between study arms, therefore the authors downgraded the level of evidence by one level.

[b] Rated "Not serious" for most outcomes due to negligible heterogeneity ($I^2 = 0\%$ or <10%), except for transient hypoxia in ALG vs. BG ($I^2 = 38.3\%$, rated "Serious").
[c] Rated "Not serious" for all outcomes, as studies directly addressed the population, interventions, and outcomes.
[d] Rated "Not serious" for minor bleeding due to significant results and narrower CIs; rated "Serious" for other outcomes due to low event rates and wide CIs crossing the null. The authors decided to rate down for imprecision.
The absolute effect was calculated using the formula: Absolute effect = Control event rate × (RR – 1) × 1000 (per 1,000).

[a*] Calculated using control (USG) event rate of 6.50% (18/277 from study data) and RR 2.30.
[b*] Calculated using control (BG) event rate of 13.93% (28/201 from study data) and RR 0.42.
[c*] Calculated using control (BG) event rate of 7.17% (16/223 from study data) and RR 1.81.
[d*] Calculated using control (USG) event rate of 1.44% (4/277 from study data) and RR 2.62.
[e*] Calculated using control (BG) event rate of 2.49% (5/201 from study data) and RR 0.46.
[f*] Calculated using control (BG) event rate of 0.45% (1/223 from study data) and RR 2.20.
[g*] Calculated using control (USG) event rate of 2.17% (6/277 from study data, assuming 0 events for studies with 0.5) and RR 1.07.
[h*] Calculated using control (BG) event rate of 4.48% (9/201 from study data) and RR 0.37.
[i*] Calculated using control (BG) event rate of 5.83% (13/223 from study data) and RR 0.82.
[j*] Calculated using control (USG) event rate of 0% (0/277 from study data, with continuity correction of 0.5/277.5) and RR 1.32.
[k*] Calculated using control (BG) event rate of 4.48% (9/201 from study data) and RR 1.25.
[l*] Calculated using control (USG) event rate of 0.72% (2/277 from study data) and RR 2.35.
[m*] Calculated using control (BG) event rate of 5.47% (11/201 from study data) and RR 0.95.
[n*] Calculated using control (BG) event rate of 0% (0/223 from study data, with continuity correction of 0.5/223.5) and RR 2.45.

Summary of Certainty Ratings:

Minor bleeding: Moderate (one downgrade for risk of bias) due to significant results, low heterogeneity, and no other concerns.
Major bleeding, Transient hypotension, Endotracheal tube cuff puncture, pneumothorax: Low (two downgrades: risk of bias, imprecision) due to low event rates and wide CIs.
Transient hypoxia (ALG vs. BG)**:** Very low (three downgrades: risk of bias, inconsistency, imprecision) due to moderate heterogeneity ($I^2 = 38.3\%$).
Transient hypoxia (ALG vs. USG, USG vs. BG)**:** Low (two downgrades: risk of bias, imprecision).

.
